# Impact of the Order of Movement on the Median Nerve Root Function: A Neurophysiological Study with Implications for Neurodynamic Exercise Sequencing

**DOI:** 10.3390/jcm13030913

**Published:** 2024-02-05

**Authors:** Dalia Ibrahim, Amal Ahbouch, Raneen Mohammed Qadah, Meeyoung Kim, Saud M. Alrawaili, Ibrahim M. Moustafa

**Affiliations:** 1 Department of Physiotherapy, College of Health Sciences, University of Sharjah, Sharjah 27272, United Arab Emirates; dhussein@sharjah.ac.ae (D.I.); aahbouch@sharjah.ac.ae (A.A.); rqadah@sharjah.ac.ae (R.M.Q.); mkim@sharjah.ac.ae (M.K.); 2 Neuromusculoskeletal Rehabilitation Research Group, RIMHS–Research Institute of Medical and Health Sciences, University of Sharjah, Sharjah 27272, United Arab Emirates; 3 Department of Physical Therapy and Health Rehabilitation, College of Applied Medical Sciences, Prince Sattam Bin Abdulaziz University, Al-Kharj 11942, Saudi Arabia; s.alrawaili@psau.edu.sa

**Keywords:** neurodynamic exercise, nerve function, dermatomal somatosensory evoked potentials, nerve root

## Abstract

**Background:** Neurodynamic exercise is a common clinical practice used to restore neural dynamic balance. The order in which movements are performed during these exercises is believed to play a crucial role in their effectiveness. This study aimed to investigate the impact of different sequences of neurodynamic exercise on nerve root function, with a specific focus on the median nerve. **Methods:** Participants were assigned randomly to three experimental groups, each undergoing a different test sequence: standard, proximal-to-distal, and distal-to-proximal. Dermatomal somatosensory evoked potentials (DSSEPs) were recorded at key levels (C6, C7, C8, and T1). **Results:** The findings revealed a significant influence of the movement sequence on DSSEP amplitudes. The execution of neurodynamic exercise in the proximal-to-distal sequence was associated with a notable reduction in amplitudes (*p* < 0.05). Conversely, the distal-to-proximal sequence resulted in increased amplitudes compared to the standard sequence (*p* < 0.05). **Conclusions:** This study underscores the importance of carefully considering the order of movements during neurodynamic exercising, particularly when evaluating nerve roots that lack the protective perineurium. The choice of sequence appears to have a substantial impact on nerve function, with implications for optimizing clinical neurodynamic exercise techniques.

## 1. Introduction

Neurodynamic techniques are frequently employed in clinical practice with a dual emphasis on either assessment, targeting the mechanosensitivity of the nervous system, or treatment, aiming to restore disrupted homeostasis in and around the nervous system [1]. In the context of treatment, these techniques can be broadly classified into two categories: ‘tensioning techniques’ and ‘sliding techniques’ [2]. While both categories share the overarching goal of mobilizing the nervous system [3] tensioning techniques involve a slightly more assertive maneuvering, leading to a notable escalation in nerve strain. Conversely, sliding techniques offer a more conservative approach, enabling the mobilization of the nervous system without significant increases in strain [1,4]. This differentiation underscores the critical importance of exercising heightened caution when employing tensioning techniques, emphasizing the necessity for precision and meticulous care in their application. Recognizing the varying impact on nerve strain between these techniques is imperative for clinicians. This recognition guides their decision-making process and ensures the judicious selection of neurodynamic interventions based on individual patient needs and considerations [5].

For neurodynamic exercise to be more effective, it requires a certain sequence [6,7]. Standardized sequences for neurodynamic exercise have been recommended to improve test reproducibility [8]. However, healthcare practitioners are also encouraged to adjust the order of movement to match an individual patient’s presentation [9].

In the context of established clinical practices, the conventional understanding is that the order in which movements are performed could affect the strain on a specific nerve segment [10]. A study has been conducted, which aimed to evaluate the effect of different movement orders on nerve biomechanics. This study focused on median nerve and simulated all three possible sequences. Unexpectedly, the order in which joint movements were executed did not show any significant effect on the final strain experienced at the endpoint or the overall longitudinal movement of the median nerve. Interestingly, a distinct observation emerged. Unlike the strain experienced at the conclusion of a test, the pattern of strain during the progressive stages of the test was notably influenced by the chosen testing sequence. This suggests that various movement sequences could potentially result in prolonged strain on the median nerve [11] Therefore, the application of different neurodynamic sequences in clinical practice is likely to be based on a biomechanical rationale, where certain sequences place a higher strain on the targeted nerve segment for a longer period, rather than a larger amount of strain at the end of the test.

Another study has highlighted that neurodynamic sequencing aids in increasing the specificity of the neurodynamic test. The test has confirmed high specificity [12].

The nerves are characterized by viscoelastic properties that exhibit time-dependent behavior, where the amount of strain, stress, deformation, and recovery rates are influenced by the duration of the applied load or stress [13]. Accordingly, prolonged strain can lead to time-dependent changes in its mechanical and neural functions [2]. Therefore, it is important to understand the time-dependent viscoelastic properties of the nerve to design and implement effective neurodynamic exercises and interventions in clinical settings [14,15].

Of interest, nerve roots, which are responsible for transmitting sensory and motor information between the spinal cord and the rest of the body, are more vulnerable to adverse neural tension because they lack the protective layer of perineurium [15,16]. The absence of perineurium makes the nerve roots particularly sensitive to a prolonged time of strain [13,17]. As a result, different sequences with different strain pattern might alter the neural functions [18]. It is, therefore, important to consider the vulnerability of nerve roots when designing and implementing neurodynamic exercises and interventions in clinical settings, to minimize the risk of adverse neural tension and optimize patient outcomes [19].

Of interest, the comparison and subsequent conclusions about appropriate sequencing in the previous studies [11] are based mainly on mechanical factors, such as strain level and amount of nerve excursion, while ignoring the neurophysiological adverse effects that may be created during different strain period.

In conclusion, mechanical loading, including tensile forces, is fundamental to maintain the homeostasis in the nervous system [20,21]. Moreover, the response is influenced by the characteristics of tensile loading, such as the magnitude, duration, rate of loading, and frequency [21,22,23]. Notably, neurodynamic exercise sequencing and the order of movement play a significant role in influencing and modulating these factors. To the authors’ knowledge, there are no studies that have investigated the effect of different sequences on the peripheral neural function [24]. Building on the findings of prior research, which have highlighted distinctive excursion patterns and strain times associated with different exercise sequences, the current study aimed to determine whether different sequences of a median nerve neurodynamic exercise could elicit varying effects on neural function [25]. The current study tests the main hypothesis that different sequences of a median nerve neurodynamic exercise will differently affect the median nerve root function.

## 2. Methods

A prospective, parallel, and randomized controlled trial was undertaken at the research laboratory affiliated with the University of Sharjah, United Arab Emirates. The trial was registered with ClinicalTrials.gov under the identifier NCT05813002, and the study protocol is publicly available. The trial was approved by the institutional research ethics committee (REC-22-06-04-S). All participants provided informed consent before data collection. Recruitment efforts were directed at the university community through printed advertisements and various social media channels. Specifically, the promotional content was tailored to engage students, alumni, and employees of the university.

### 2.1. Participants

The study included individuals of both genders, aged between 18 and 30, who were in good health and had not experienced any neck or arm pain in the past year (Table 1). Rigorous physical and neurological assessments were conducted by a comprehensive examination of their medical records, ensuring the absence of any red flags. The researchers aimed to ensure homogeneity among participants in terms of health status, and therefore, individuals with the following conditions were checked for and excluded: (i) a disease that causes inflammation in the joints or other parts of the body; and (ii) a history of serious injury or surgery that affected the muscles, bones, or joints or any condition that affected the spine or limbs.

The study incorporated three groups of participants who were subjected to varied neurodynamic exercise sequences. Participants were randomly assigned to one of the three groups:

- Standard neurodynamic sequence: this group received the standard exercise for median nerve entrapment [26];
- Distal-to-proximal neurodynamic sequence (DTP): this group received a treatment that involved moving the median nerve from the distal end (near the hand) to the proximal end (near the shoulder);
- Proximal-to-distal neurodynamic sequence (PTD): this group received a treatment that involved moving the median nerve from the proximal end to the distal end.

The randomization process was based on permuted blocks of variable sizes. Each randomly generated permuted block was assigned a sequential number and placed in opaque sealed envelopes, which were securely stored until needed. Upon participant inclusion, the researcher opened the next envelope in the sequence in the participant’s presence, determining group assignment (where the participant would still be blinded to the group they were assigned to). Data concerning participants’ age, weight, and smoking status were collected. In the standard group, participants’ age ranged from 19 to 25, with weights between 61 and 73 kg. The group consisted of 13 males and 17 females, including 19 nonsmokers and 11 smokers. In the DTP group, participants’ age ranged from 17 to 25, with weights ranging from 57 to 71 kg. The group comprised 12 males and 8 females, with 20 nonsmokers and 10 smokers In the PTD group, participants’ age ranged from 21 to 25, with weights between 63 and 73 kg. The group included 14 males and 16 females, with 17 nonsmokers and 13 smokers.

### 2.2. Interventions

#### 2.2.1. Standard Neural Tensioning Mobilization

For this technique, joint movements were performed to increase the length of the nerve bed, while allowing tensile loading to the median nerve. With the participant in the supine lying position, the shoulder girdle was depressed, the glenohumeral joint was abducted (110°) and laterally rotated, the wrist and fingers were extended, the forearm was supinated, and then the elbow was extended [26]. The therapist executed ten elbow flexion/extension movements while maintaining the intervention position for the rest of the joints. Four sets of ten tensioning movements were performed at a rhythm of approximately 6 s per cycle, followed by a 1 min rest between each series. After each cycle of 10 repetitions, the position was held for 10 s. Participants were blinded to the specific neuromobilization (NM) technique administered during the intervention.

#### 2.2.2. Proximal-to-Distal Neural Tensioning Mobilization

**Figure 1:**
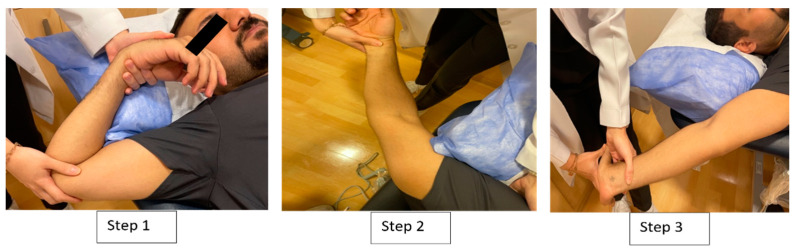
Proximal-to-distal median nerve neural tensioning mobilization.

The participants in this group received the proximal-to-distal sequence, which started with shoulder depression, abduction, and lateral rotation. The forearm was supinated, followed by the extension of the elbow, wrist, and fingers [27]. The practitioner conducted ten movements of wrist flexion and extension, maintaining the stability of the testing position for the other joints. Sets of ten tension-inducing motions were carried out four times, with each cycle lasting around 6 s and a rest period of 1 min separating each set. After each set of 10 repetitions, the position was maintained for 10 s (Figure 1).

#### 2.2.3. Distal-to-Proximal Neural Tensioning Mobilization

**Figure 2:**
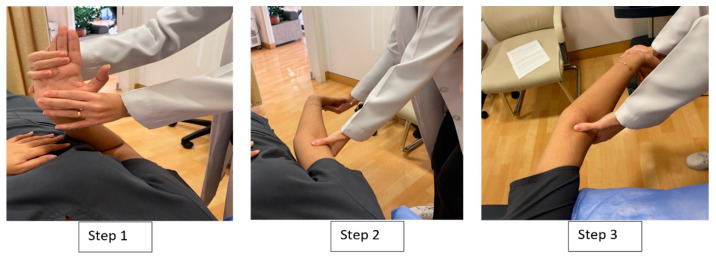
Distal-to-proximal median nerve neural tensioning mobilization.

The participants in this group received the distal-to-proximal sequence, which started with wrist and fingers’ extension, progressed to forearm supination and extension, and ended with shoulder depression, abduction, and external rotation [28]. The therapist performed 10 shoulder abduction/adduction movements while maintaining the test position for rest of the joints. Four series of 10 tensioning movements, with a rhythm of approximately 6 s per cycle and a 1 min rest between each series, were performed. After each cycle of 10 repetitions, the position was held for 10 s (Figure 2).

The intervention program was administered on a one-on-one basis by a physiotherapist with 5 years of clinical experience and specialized training in these techniques. To minimize variability between therapists, ensure consistency, and maintain a consistent patient–therapist relationship, the same therapist treated all groups.

While the therapist administering the treatment was aware of the therapeutic approach employed, the participants and the assessor evaluating the results were kept uninformed about how the participants were randomly assigned to specific groups. Furthermore, both the participants and the outcome assessor were explicitly instructed not to engage in conversations regarding the treatment received during outcome assessments. This precaution was taken to uphold the blinding of the study and mitigate the possibility of any biases.

### 2.3. Outcome Measures

Outcome measurements were assessed at three distinct time points: baseline, immediately post treatment session, and at the 24 h follow-up. The objective of the third evaluation was to detect any short-term carryover effects occurring within the 24 h following the intervention period. Dermatomal somatosensory evoked potentials (DSSEPs) were the primary outcome measure employed to assess the impact of the treatment. The higher the DSSEPs amplitude the better the implication it has for the neural effect [29].

Dermatomal Somatosensory Evoked Potentials (DSSEPs)

In this study, neurophysiological measurements were conducted to assess the peak-to-peak amplitude of DSSEPs at the C6, C7, C8, and T1 levels. For this purpose, an electromyography device (Neurosoft eight channel EMG–NCV machine, Ivanovo, Russia) was utilized to capture these variables.

The DSSEPs were induced by administering repetitive square wave electrical pulses (0.50 ms) with a frequency of 3 Hz. These pulses were administered using standard gel surface electrodes positioned over the cervical dermatomes. Stimulation was performed using bipolar surface electrodes with an interelectrode distance of 2 cm, placing the cathode in the proximal region. The intensity of the stimulus was adjusted to a level three times higher than each participant’s perception threshold. Throughout the testing procedure, all participants were in a supine position with their eyes closed, and they were instructed to maintain a state of calm relaxation to ensure consistency. For recording, the disc electrodes were placed at C 4′ and C 3′ which are located between C 4 and P 4, and C 3 and P 3, respectively. The reference electrode was situated at Fz, while the ground electrode was positioned at Fpz. The electrodes’ impedance was kept below 5 kΩ. Carefully chosen dermatome stimulation locations were chosen so as not to stimulate major nerve trunks, in accordance with recognized clinical and anatomical understanding of dermatome borders. During each session, two full recording sequences were performed for all stimulated dermatomes, spanning from C6 to T1. This yielded an average of 500 cortical responses recorded from the recording electrodes on the opposite side of the scalp in accordance with the approach detailed by Coppieters and Alshami in 2007 [26]. For the DSSEPs, a higher amplitude indicates less adverse neural effect.

### 2.4. Sample Size Determination

To determine the required number of participants for the trial, the mean and standard deviation estimates were obtained from a pilot trial involving 10 participants who underwent the same program. The mean differences and standard deviations of the peak-to-peak amplitude of DSSEPs were recorded for different levels, namely C6, C7, C8, and T1. The mean differences (± SD) were observed as follows: C6 (−0.29 ± 0.40 µV), C7 (−0.10 ± 0.50 µV), C8 (−0.30 ± 0.60 µV), and T1 (−0.40 ± 0.60 µV).

Using these values, the sample size was estimated separately for each primary outcome, while adjusting the significance level with the Bonferroni method. The largest value obtained from these calculations determined the optimal sample size for the study, which indicated a minimum of 27 participants in each group, with a statistical power of 80%. To account for potential dropouts, the sample size was increased by 10%.

### 2.5. Data Analysis

The normality of the distribution for all collected data was examined using the Kolmogorov–Smirnov test. Additionally, the equality of variance was evaluated through Levene’s test [30]. To achieve a 95% confidence level, the significance level (*p*-value) was set at <0.05. One-way ANOVA was employed to assess the between-group differences in continuous variables, whereas the χ^2^ test was used for categorical variables.

For comparing the outcome measures between groups, a 2-way repeated-measures analysis of covariance was applied. The model incorporated one independent factor (groups), one repeated measure (three time-points: pre-intervention, post-intervention, and 24 h follow-up), and an interaction factor (group × time). If significant interactions were observed (*p* < 0.05), post hoc analysis with Bonferroni correction was conducted.

To account for between-group differences, the baseline values of DSSEPs were used as covariates. The baseline covariates were centered by subtracting each participants score value from the overall mean.

## 3. Results

### 3.1. Participant Flow and Characteristics

**Figure 3:**
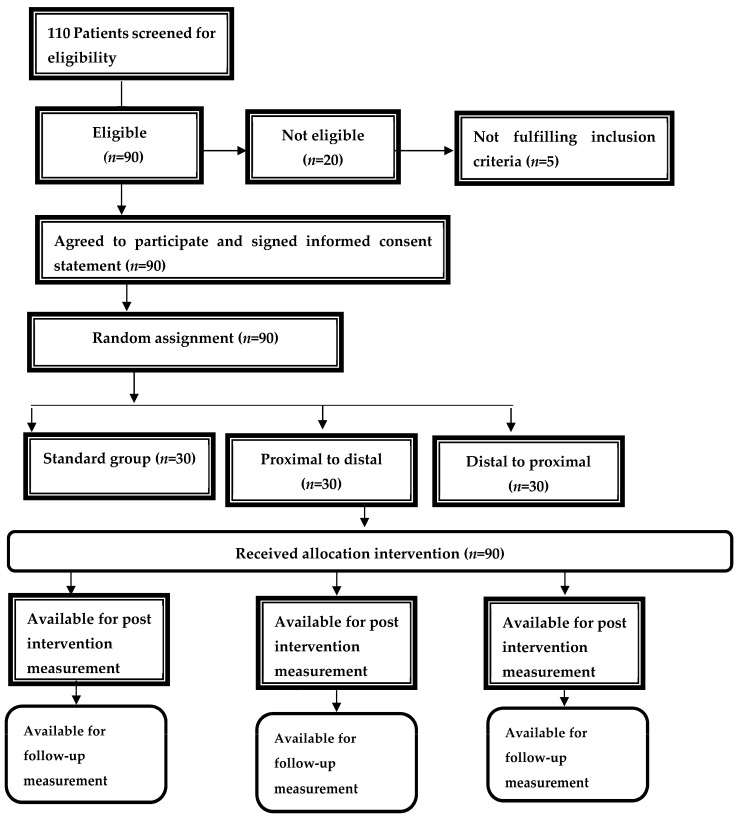
Flowchart of participants’ randomization and retention throughout the study.

Figure 3 provides a comprehensive visualization of the participants randomization and retention throughout the study. Initially, the sample consisted of 110 volunteers. Among these, five participants (4.50% of the initial sample) were found ineligible due to non-compliance with inclusion criteria or meeting exclusion criteria, and fifteen participants (13.60%) opted not to participate. Ultimately, 90 participants met the study’s inclusion criteria and successfully concluded the entire study protocol. The baseline characteristics across all three groups demonstrated parity, with no significant differences observed (Table 1).

### 3.2. Neurophysiological Outcomes

The analysis utilizing a two-way repeated measures ANOVA exposed a significant interaction effect, highlighting the synergy of group and time on DSSEPs at the C6, C7, C8, and T1 levels (*p* < 0.001). For a detailed breakdown of these outcomes, refer to Table 2 and Table 3.

**Figure 4:**
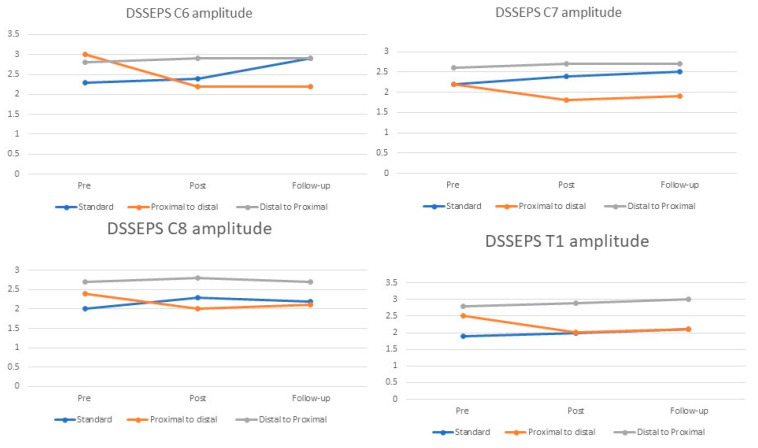
DSSEP amplitudes of C6-T1 at three specific time points: baseline, immediately after the treatment session, and at 24 h follow-up.

Further analysis of the PTD group revealed a notable decline in DSSEP amplitudes at the C6, C7, C8, and T1 levels, amounting to 27%, 27%, 29%, and 16%, respectively (*p* < 0.05) compared to the baseline measurements (Figure 4). This reduction was observed when comparing baseline measurements with post-intervention measurements. In contrast, during the same period, the DTP group exhibited an increase in DSSEP amplitudes at C6, C7, C8, and T1 by 7%, 44%, 27%, and 27%, respectively. Similarly, the Standard group demonstrated amplitude increments in DSSEPs at C6, C7, C8, and T1 by 4%, 6%, 0%, and 1%, respectively. These specific changes and their magnitudes can be found in greater details in Table 2 and Table 3.

In the follow-up assessments, the PTD group exhibited a decrement in DSSEP amplitude at C6, C7, C8, and T1 of 0%, 3%, 4%, and 5%, respectively, relative to the initial measurement. On the contrary, the DTP group displayed an elevation of 0%, 6%, 24%, and 6%, respectively, over the same interval. The amplitude within the Standard group underwent a decrease ranging from 0% to 4%. Further insights can be obtained from the data presented in Table 2 and Table 3.

## 4. Discussion

The primary objective of the current study was to investigate the impact of various neurodynamic exercise sequences on nerve root function, with a specific focus on the median nerve. The primary outcome measure was the DSSEPs recorded at different levels of the nerve, namely C6, C7, C8, and T1.

Neurodynamic techniques are commonly used in clinical practice to normalize nerve mobility and decrease mechanical loads on the nervous system [21]. Previous studies have shown that neurodynamic techniques can increase nerve mobility and reduce fibrous adhesions between neural tissues [31]. However, the order of movements in neurodynamic exercising is considered crucial for their effectiveness, with standardized sequences recommended for better test reproducibility [32]. Nonetheless, clinicians are also encouraged to adapt the sequence based on individual patient presentations [33].

In our study, the decision to focus on the tensioning technique over the gliding technique was rooted in a careful consideration of safety and potential risks. After a comprehensive review of the existing literature, we determined that the tensioning technique offered a less controlled approach with high risk factors compared to the gliding technique. The tensioning technique increase the potential for nerve adverse reactions [29]. Our decision was further supported by a desire to gather comprehensive data on the effects of neural mobilization on peripheral nervous system function while mitigating the potential for adverse events.

The current study demonstrated that different neurodynamic sequences had varying effects on nerve root function as measured by DSSEPs. The three experimental groups, each subjected to a different sequence of neurodynamic exercising, showed distinct changes in DSSEPs at different time points. The PTD sequence resulted in a significant decrease in DSSEP amplitudes for C6, C7, C8, and T1, while the DTP sequence led to increases in DSSEP amplitudes at the same nerve levels. On the other hand, the standard sequence showed minimal changes in DSSEP amplitudes.

These findings suggest that the order of movements in neurodynamic exercising can influence the strain pattern and excursion of the nerve segment, which in turn may subtly impair neural function. Specifically, the PTD sequence led to a longer period of strain on the proximal part of the median nerve, potentially resulting in adverse neural tension. Nerve roots, lacking perineurium, are particularly vulnerable to prolonged strain [34]. Therefore, understanding the time-dependent viscoelastic properties of the nerve is crucial in designing effective neurodynamic tests to minimize the risk of adverse neural tension and optimize patient outcomes [35,36].

The study’s results are concordant with previous research, which has also highlighted the importance of considering the neural adverse mechanical tension that may be created during the strain period [34]. In the context of neurodynamic exercising, this becomes even more critical when dealing with nerve roots, which lack the protective perineurium and are susceptible to adverse neural tension [21]. While the mechanical factors like strain level and nerve excursion have been primarily focused on in previous studies, the current research emphasizes the significance of assessing neural function during neurodynamic exercising [37].

The specificity of the technique used in neurodynamic testing and mobilization plays a crucial role in its effectiveness, as highlighted by several studies. For instance, the research by Alshami and Bamhair on cervical vertebral mobilization for chronic cervical radiculopathy emphasizes the benefits of specific manual therapies combined with exercise [38]. This study underscores the importance of tailoring neurodynamic approaches to improve sensory features and reduce mechanical pain hypersensitivity. Similarly, the validity of upper limb neurodynamic tests (ULNTs) for detecting peripheral neuropathic pain, as discussed in another study, further stresses the need for specific and accurate diagnostic testing [32]. This is essential for ensuring the effectiveness of neurodynamic tests and for correctly interpreting their outcomes. Furthermore, the investigation into the dispersion of intraneural fluid in cervical nerve roots by Burgess et al. demonstrates that specific mobilization techniques can significantly influence internal fluid dynamics within nerve root [39]. This finding corroborates with the broader understanding that the precision of the movement sequence and technique in neurodynamic testing can have substantial effects on nerve root function. Additionally, studies by Khademi et al. and Zaheer et al. on median nerve stiffness in carpal tunnel syndrome patients reveal that immediate effects of neurodynamic mobilization are closely tied to the specific techniques employed [40]. These studies collectively highlight the critical importance of technique specificity in neurodynamic testing and mobilization, emphasizing the need for a nuanced and carefully tailored approach, which is vital for optimizing nerve function and clinical outcomes.

The findings from this study have clinical implications for the design and implementation of neurodynamic exercising and interventions. Clinicians should be cautious when selecting the sequence of movements during neurodynamic exercising, considering the potential impact on nerve root function. The DTP sequence resulted in significant reductions in DSSEP amplitudes, suggesting the possibility of adverse neural effects. Therefore, adopting alternative sequences, such as the PTD sequence, could be more appropriate to mitigate adverse neural tension.

In this study, there were participants that were smokers. Ezzati and Lopez’s comprehensive epidemiological investigation, revealed a positive association between cigarette smoking and the risk of ND. In recent research, numerous studies have delved into the impact of smoking on cognitive functions, with the majority indicating a decline in cognitive function attributed to the effects of exposure to cigarette smoke [41].

While this study sheds light on the impact of different neurodynamic sequences on nerve root function, it is essential to acknowledge its limitations. The study focused on the median nerve, and the results may not be directly applicable to other nerves. Additionally, the study was conducted on healthy young individuals, and the findings may not fully represent the responses seen in individuals with nerve-related pathologies.

In conclusion, the current study demonstrates that the order of movements in neurodynamic exercise can influence nerve root function, as measured by DSSEPs. The DTP sequence resulted in a significant decrease in DSSEP amplitudes, while the PTD sequence led to increases in DSSEP amplitudes. These findings highlight the importance of considering the vulnerability of nerve roots to adverse neural tension during neurodynamic exercising. The higher the DSSEP amplitude the better response for the neural tension. Clinicians should carefully select the sequence of movements to optimize patient outcomes and minimize the risk of adverse neural effects. Further research in different patient populations and other nerves is needed to validate and expand on these findings.

## 5. Conclusions

This research explored the impact of different neurodynamic exercise sequences on nerve root function, with a focus on the median nerve. The study revealed that the sequence of movements employed in neurodynamic exercise significantly influenced nerve root response, as measured by DSSEP amplitudes. Notably, the proximal-to-distal sequence led to decreased amplitudes, possibly indicating adverse neural tension, while the distal-to-proximal sequence induced increased amplitudes. These findings highlight the significance of selecting appropriate movement sequences during neurodynamic exercise to optimize patient outcomes and minimize the risk of adverse neural effects.

## Figures and Tables

**Table 1:** Baseline participants’ demographics and clinical information; DTP: distal-to-proximal; PTD: proximal-to-distal. χ^2^ = chi-square value. ANOVA test to compare the continuous variables and the chi-square test for categorical variables were used. Values are expressed as means ± standard deviation where indicated.

	Standard Group (*n* = 30)	DTP Group (*n* = 30)	PTD Group (*n* = 30)	*p* Value
Age (Yrs)	22 ± 3	21 ± 4	23 ± 2	F = 2.86 *p* = 0.06
Weight (kg)	67 ± 6	64 ± 7	68 ± 5	F = 2.61 *p* = 0.07
Gender				
Male	13	12	14	χ² = 0.27 *p* = 0.80
Female	17	18	16	
Smoking status				
Nonsmoker	19	20	17	χ² = 0.66 *p* = 0.71
Smoker	11	10	13	
C6 (µV)	2.30 ± 0.60	3.00 ± 0.70	2.80 ± 0.80	<0.001
C7 (µV)	2.20 ± 0.50	2.20 ± 0.30	2.60 ± 0.60	<0.001
C8 (µV)	2.00 ± 0.30	2.40 ± 0.50	2.70 ± 0.40	<0.001
T1 (µV)	1.90 ± 0.30	2.50 ± 0.50	2.80 ± 0.40	<0.001

**Table 2:** Two-way repeated ANOVA (DSSEPs). Data are expressed as mean ± standard deviation. G = group; T = time; Pre = Baseline; Post = after treatment session; Follow = 24 h follow up.

		Pre	Post	Follow	*p*		
					G	T	G vs. T
C6 (µV)	Standard	2.30 ± 0.60	2.40 ± 0.50	2.90 ± 0.50	<0.001	<0.001	<0.001
	PTD	3.00 ± 0.70	2.20 ± 0.40	2.20 ± 0.50			
	DTP	2.80 ± 0.80	2.90 ± 0.50	2.90 ± 0.70			
C7 (µV)	Standard	2.20 ± 0.50	2.40 ± 0.50	2.50 ± 0.70	<0.001	<0.001	<0.001
	PTD	2.20 ± 0.30	1.80 ± 0.60	1.90 ± 0.50			
	DTP	2.60 ± 0.60	2.70 ± 0.40	2.70 ± 0.70			
C8 (µV)	Standard	2.00 ± 0.30	2.30 ± 0.60	2.20 ± 0.70	<0.001	<0.001	<0.001
	PTD	2.40 ± 0.50	2.00 ± 0.30	2.10 ± 0.40			
	DTP	2.70 ± 0.40	2.80 ± 0.30	2.70 ± 0.80			
T1 (µV)	Standard	1.90 ± 0.30	2.00 ± 0.90	2.10 ± 0.50	<0.001	<0.001	<0.001
	PTD	2.50 ± 0.50	2.01 ± 0.60	2.10 ± 0.60			
	DTP	2.80 ± 0.40	2.90 ± 0.70	3.00 ± 0.60			

**Table 3:** Post hoc analysis matrix. * = statistically significant difference.

(I) Groups		(J) Groups	Mean Difference (I–J)	Std. Error	Sig.	95% Confidence Interval	
						Lower Bound	Upper Bound
C6 (µV)	Standard	DTP	0.50 *	0.15	0.002	−0.50	0.25
		PTD	0.70 *	0.14	0.010	0.08	0.84
C7 (µV)	Standard	DTP	−0.47 *	0.14	0.005	−0.83	−0.11
		PTD	0.70 *	0.18	0.001	0.25	1.15
C8 (µV)	Standard	DTP	−0.62 *	0.12	0.000	−0.93	−0.31
		PTD	0.45 *	0.12	0.002	0.14	0.76
T1 (µV)	Standard	DTP	−0.16	0.10	0.400	−0.45	0.11
		PTD	0.40 *	0.11	0.002	0.12	0.69

## Data Availability

The datasets analyzed in the current study are available from the corresponding author upon reasonable request.
